# Crystal structure of 2-cyano-*N*-(furan-2-ylmeth­yl)acetamide

**DOI:** 10.1107/S2056989015010488

**Published:** 2015-06-10

**Authors:** Shivanna Subhadramma, Budanur Papaiah Siddaraju, Chandra Naveen, Janardhanan Saravanan, Dasararaju Gayathri

**Affiliations:** a Department of Physics, Vijaya College, Basavanagudi, Bangalore 560 004, India; b Department of Engineering Chemistry, Cauvery Institute of Technology, Sundhahalli, Mandya, India; c Department of Chemistry, Post-Graduate and Research Centre, St. Joseph’s College (Autonomous), Bangalore 560 027, India; d Department of Pharmaceutical Chemistry, PES College of Pharmacy, Hanumanthnagar, Bangalore 560 050, India; e Centre of Advanced Study in Crystallography and Biophysics, University of Madras, Guindy Campus, Chennai 600 025, India

**Keywords:** crystal structure, furan, acetamide, cyano, bifurcated hydrogen bonding.

## Abstract

In the title compound, C_8_H_8_N_2_O_2_, the acetamide unit is inclined to the furan ring by 76.7 (1)°. In the crystal, mol­ecules are linked by N—H⋯O and C—H⋯O hydrogen bonds, generating *C*(4) chains along [100]. The carbonyl O atom is a bifurcated acceptor and an *R* ^1^_2_(6) ring is formed.

## Related literature   

For examples of biological properties of furan derivatives, see: Anupam *et al.* (2011 ▸). For the biological activities of some heterocyclic derivatives containing the acetamide moiety, see: Fallah-Tafti *et al.* (2011 ▸); Shams *et al.* (2011 ▸). For a related acetamide structure, see: Jasinski *et al.* (2013 ▸). For the crystal structure of similar compound, 2-cyano-*N*-furfuryl-3-(2-fur­yl)acryl­amide, see: Pomés Hernández *et al.* (1996 ▸).
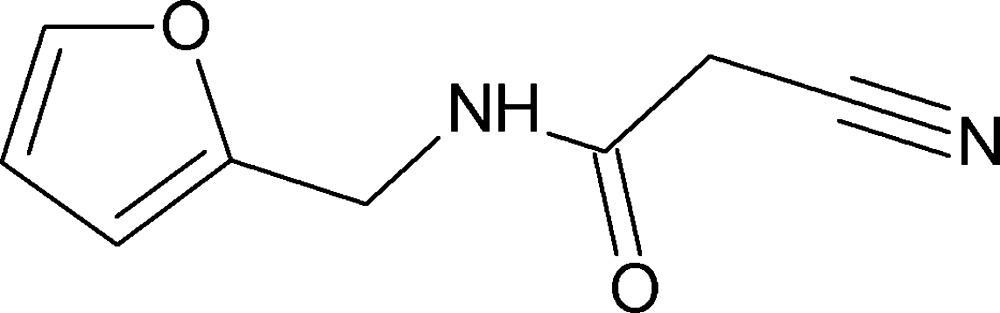


## Experimental   

### Crystal data  

C_8_H_8_N_2_O_2_
*M* *_r_* = 164.16
Monoclinic, 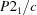

*a* = 4.8093 (4) Å
*b* = 14.9495 (16) Å
*c* = 11.4969 (11) Å
β = 93.482 (3)°
*V* = 825.06 (14) Å^3^
*Z* = 4
Mo *K*α radiation
μ = 0.10 mm^−1^
*T* = 293 K
0.3 × 0.2 × 0.2 mm

### Data collection  

Bruker APEXII CCD diffractometer
Absorption correction: multi-scan (*SADABS*; Bruker, 2004 ▸) *T*_min_ = 0.946, *T*_max_ = 0.986
7302 measured reflections
1455 independent reflections
1175 reflections with *I* > 2σ(*I*)
*R*_int_ = 0.027

### Refinement  

*R*[*F* ^2^ > 2σ(*F* ^2^)] = 0.037
*wR*(*F* ^2^) = 0.111
*S* = 1.09
1455 reflections
109 parameters
H-atom parameters constrained
Δρ_max_ = 0.14 e Å^−3^
Δρ_min_ = −0.13 e Å^−3^

### 

Data collection: *APEX2* (Bruker, 2004 ▸); cell refinement: *APEX2* and *SAINT* (Bruker, 2004 ▸); data reduction: *SAINT* and *XPREP* (Bruker, 2004 ▸); program(s) used to solve structure: *SHELXS2014/7* (Sheldrick, 2008 ▸); program(s) used to refine structure: *SHELXL2014*/7 (Sheldrick, 2015 ▸); molecular graphics: *PLATON* (Spek, 2009 ▸); software used to prepare material for publication: *SHELXL2014/7* and *PLATON*.

## Supplementary Material

Crystal structure: contains datablock(s) I, globa. DOI: 10.1107/S2056989015010488/su5145sup1.cif

Structure factors: contains datablock(s) I. DOI: 10.1107/S2056989015010488/su5145Isup2.hkl

Supporting information file. DOI: 10.1107/S2056989015010488/su5145Isup3.cml

**Fig. 1.**
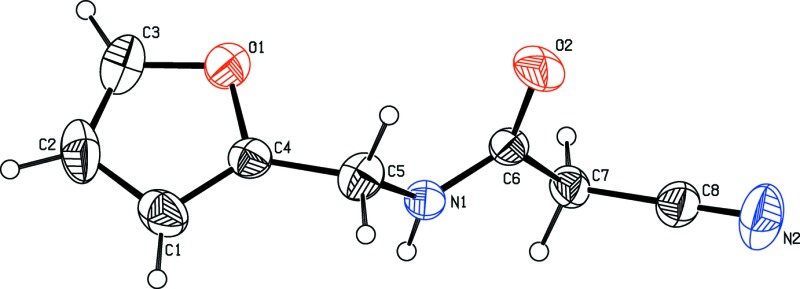
The molecular structure of the title compound, with atom labelling. The displacement ellipsoids are drawn at the 30% probability level.

**Fig. 2.**
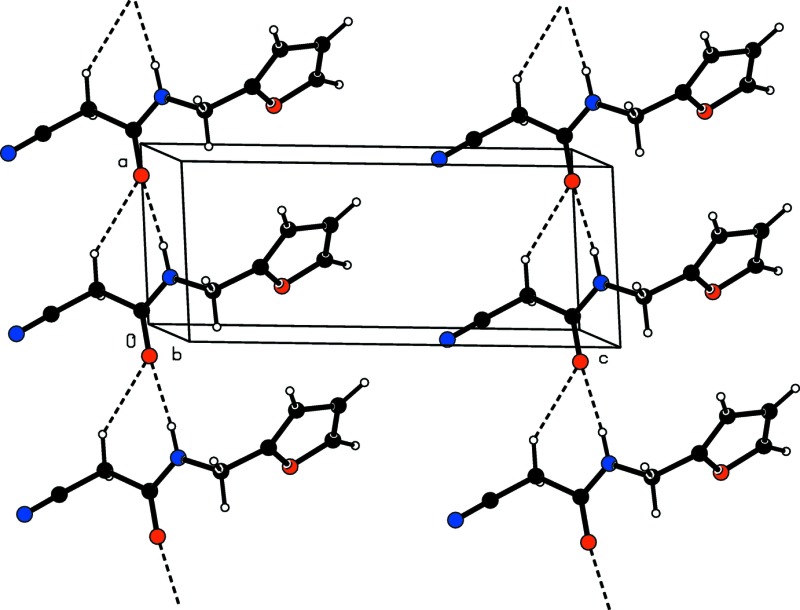
A view along the b axis of the crystal packing of the title compound. The hydrogen bonds are shown as dashed lines (see Table 1 for details).

. DOI: 10.1107/S2056989015010488/su5145fig1.tif
The mol­ecular structure of the title compound, with atom labelling. The displacement ellipsoids are drawn at the 30% probability level.

b . DOI: 10.1107/S2056989015010488/su5145fig2.tif
A view along the *b* axis of the crystal packing of the title compound. The hydrogen bonds are shown as dashed lines (see Table 1 for details).

CCDC reference: 1404031

Additional supporting information:  crystallographic information; 3D view; checkCIF report

## Figures and Tables

**Table 1:** Hydrogen-bond geometry (, )

*D*H*A*	*D*H	H*A*	*D* *A*	*D*H*A*
N1H1O2^i^	0.86	1.99	2.846(1)	175
C7H7*A*O2^i^	0.97	2.55	3.395(2)	145

Symmetry code: (i) 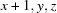.

## supplementary crystallographic information

### S1. Structural commentary

Furan derivatives are gaining importance for their wide pharmacological activities like anti­bacterial, anti­tumor, anti-inflammatory, anti­fungal, and analgesic (Anupam *et al.*, 2011). Acetamide derivatives have been shown to possess various biological properties, and recently, the synthesis and biological activities of some heterocyclic derivatives containing the acetamide moiety have been reported (Fallah-Tafti *et al.*, 2011; Shams *et al.*, 2011). In continuation of our work on the synthesis of acetamide derivatives (Jasinski *et al.*, 2013), we report herein on the synthesis and crystal structure of the title compound.

The title molecule, Fig. 1, is Z-shaped. The furan ring (O1/C1—C4) is nearly perpendicular with the mean plane of the acetamide group (O2/N1/C6/C7) with a dihedral angle of 76.7 (1)°. The aceto­nitrile moiety is inclined to the mean plane of the acetamide group by 54 (6) °. The bond lengths and angles are close to those reported for a very similar structure, 2-cyano-*N*-furfuryl-3-(2-furyl)acryl­amide (Pomés Hernández *et al.*, 1996).

The crystal packing is stabilized by N—H···O and C—H···O hydrogen bonds (Table 1 and Fig. 2). Atoms N1 and C7 act as donors to a bifurcated acceptor O-atom, O2, generating C(4) chains along the *a*-axis and, as a consequence, an *R*_2_^1^(6) ring is formed.

### S2. Synthesis and crystallization

An equimolar mixture of furfuryl amine and ethyl cyano acetate were mixed in a conical flask and the mixture was heated under microwave irradiation at 700 W for 3 min with an inter­val of 20 seconds each time. The mixture was then poured to a beaker and cooled giving a solid whose size reduced, washed with ethanol. It was recrystallized from an acetone/water mixture (7:3), yielding colourless block-like crystals on slow evaporation of the solvent.

### S3. Refinement

Crystal data, data collection and structure refinement details are summarized in Table 2. The NH and C-bound H atoms were included in calculated positions and refined using a riding model: N—H = 0.86 Å, C—H = 0.93 - 0.97 Å with *U*_iso_(H) = 1.2*U*_eq_ (N,C).

### Crystal data

**Table:**

C_8_H_8_N_2_O_2_	*F*(000) = 344
*M**_r_* = 164.16	*D*_x_ = 1.322 Mg m^−^^3^
Monoclinic, *P*2_1_/*c*	Mo *K*α radiation, λ = 0.71073 Å
*a* = 4.8093 (4) Å	Cell parameters from 1455 reflections
*b* = 14.9495 (16) Å	θ = 2.2–25.0°
*c* = 11.4969 (11) Å	µ = 0.10 mm^−^^1^
β = 93.482 (3)°	*T* = 293 K
*V* = 825.06 (14) Å^3^	Block, colourless
*Z* = 4	0.3 × 0.2 × 0.2 mm

### Data collection

**Table:**

Bruker APEXII CCD diffractometer	1455 independent reflections
Radiation source: fine-focus sealed tube	1175 reflections with *I* > 2σ(*I*)
Graphite monochromator	*R*_int_ = 0.027
Detector resolution: 8.0216 pixels mm^-1^	θ_max_ = 25.0°, θ_min_ = 2.2°
ω and φ scan	*h* = −5→5
Absorption correction: multi-scan (*SADABS*; Bruker, 2004)	*k* = −17→17
*T*_min_ = 0.946, *T*_max_ = 0.986	*l* = −12→13
7302 measured reflections	

### Refinement

**Table:**

Refinement on *F*^2^	0 restraints
Least-squares matrix: full	Hydrogen site location: inferred from neighbouring sites
*R*[*F*^2^ > 2σ(*F*^2^)] = 0.037	H-atom parameters constrained
*wR*(*F*^2^) = 0.111	*w* = 1/[σ^2^(*F*_o_^2^) + (0.0542*P*)^2^ + 0.1342*P*] where *P* = (*F*_o_^2^ + 2*F*_c_^2^)/3
*S* = 1.09	(Δ/σ)_max_ < 0.001
1455 reflections	Δρ_max_ = 0.14 e Å^−^^3^
109 parameters	Δρ_min_ = −0.13 e Å^−^^3^

### Special details

**Table:**

Geometry. All e.s.d.'s (except the e.s.d. in the dihedral angle between two l.s. planes) are estimated using the full covariance matrix. The cell e.s.d.'s are taken into account individually in the estimation of e.s.d.'s in distances, angles and torsion angles; correlations between e.s.d.'s in cell parameters are only used when they are defined by crystal symmetry. An approximate (isotropic) treatment of cell e.s.d.'s is used for estimating e.s.d.'s involving l.s. planes.

### Fractional atomic coordinates and isotropic or equivalent isotropic displacement parameters (Å^2^)

**Table:**

	*x*	*y*	*z*	*U*_iso_*/*U*_eq_	
C1	0.6009 (4)	0.71318 (14)	0.26789 (17)	0.0705 (5)	
H1A	0.7123	0.7616	0.2502	0.085*	
C2	0.6246 (4)	0.65991 (17)	0.37051 (16)	0.0816 (6)	
H2	0.7549	0.6667	0.4330	0.098*	
C3	0.4271 (5)	0.59926 (15)	0.36003 (17)	0.0777 (6)	
H3	0.3942	0.5558	0.4154	0.093*	
C4	0.3883 (3)	0.68024 (10)	0.20223 (13)	0.0483 (4)	
C5	0.2563 (3)	0.70388 (11)	0.08709 (13)	0.0575 (4)	
H5A	0.0559	0.7057	0.0925	0.069*	
H5B	0.3170	0.7633	0.0660	0.069*	
C6	0.1307 (3)	0.59915 (10)	−0.07049 (12)	0.0443 (4)	
C7	0.2372 (3)	0.54182 (11)	−0.16707 (12)	0.0506 (4)	
H7A	0.4373	0.5489	−0.1694	0.061*	
H7B	0.1983	0.4793	−0.1522	0.061*	
C8	0.1019 (4)	0.56845 (11)	−0.27806 (15)	0.0590 (4)	
N1	0.3219 (2)	0.64139 (9)	−0.00467 (10)	0.0467 (3)	
H1	0.4942	0.6316	−0.0164	0.056*	
N2	−0.0079 (5)	0.59089 (12)	−0.36283 (16)	0.0947 (6)	
O1	0.2779 (2)	0.60909 (8)	0.25698 (10)	0.0649 (4)	
O2	−0.1197 (2)	0.60352 (9)	−0.05760 (11)	0.0713 (4)	

### Atomic displacement parameters (Å^2^)

**Table:**

	*U*^11^	*U*^22^	*U*^33^	*U*^12^	*U*^13^	*U*^23^
C1	0.0617 (10)	0.0790 (13)	0.0714 (12)	−0.0144 (9)	0.0087 (9)	−0.0277 (10)
C2	0.0772 (13)	0.1118 (17)	0.0535 (11)	0.0191 (13)	−0.0147 (9)	−0.0260 (11)
C3	0.1019 (15)	0.0806 (14)	0.0500 (10)	0.0116 (12)	−0.0012 (10)	−0.0002 (9)
C4	0.0465 (8)	0.0503 (9)	0.0491 (8)	0.0010 (7)	0.0112 (6)	−0.0087 (7)
C5	0.0624 (10)	0.0576 (10)	0.0535 (9)	0.0132 (8)	0.0110 (7)	−0.0042 (7)
C6	0.0312 (7)	0.0565 (9)	0.0455 (8)	0.0070 (6)	0.0038 (6)	0.0080 (7)
C7	0.0404 (7)	0.0614 (10)	0.0497 (9)	0.0038 (7)	0.0004 (6)	−0.0016 (7)
C8	0.0713 (11)	0.0528 (10)	0.0526 (10)	−0.0083 (8)	0.0015 (8)	0.0013 (8)
N1	0.0339 (6)	0.0611 (8)	0.0458 (7)	0.0072 (5)	0.0079 (5)	−0.0030 (6)
N2	0.1407 (18)	0.0789 (12)	0.0611 (10)	−0.0056 (11)	−0.0226 (11)	0.0125 (9)
O1	0.0713 (8)	0.0667 (8)	0.0568 (7)	−0.0098 (6)	0.0037 (6)	0.0023 (6)
O2	0.0304 (6)	0.1024 (10)	0.0816 (9)	0.0076 (6)	0.0088 (5)	−0.0081 (7)

### Geometric parameters (Å, º)

**Table:**

C1—C4	1.328 (2)	C5—H5A	0.9700
C1—C2	1.422 (3)	C5—H5B	0.9700
C1—H1A	0.9300	C6—O2	1.2238 (16)
C2—C3	1.314 (3)	C6—N1	1.3162 (18)
C2—H2	0.9300	C6—C7	1.516 (2)
C3—O1	1.355 (2)	C7—C8	1.452 (2)
C3—H3	0.9300	C7—H7A	0.9700
C4—O1	1.3602 (19)	C7—H7B	0.9700
C4—C5	1.476 (2)	C8—N2	1.131 (2)
C5—N1	1.4579 (19)	N1—H1	0.8600
			
C4—C1—C2	106.51 (18)	C4—C5—H5B	108.9
C4—C1—H1A	126.7	H5A—C5—H5B	107.7
C2—C1—H1A	126.7	O2—C6—N1	124.26 (14)
C3—C2—C1	106.82 (17)	O2—C6—C7	119.83 (13)
C3—C2—H2	126.6	N1—C6—C7	115.90 (12)
C1—C2—H2	126.6	C8—C7—C6	109.59 (13)
C2—C3—O1	110.29 (18)	C8—C7—H7A	109.8
C2—C3—H3	124.9	C6—C7—H7A	109.8
O1—C3—H3	124.9	C8—C7—H7B	109.8
C1—C4—O1	109.60 (15)	C6—C7—H7B	109.8
C1—C4—C5	134.05 (17)	H7A—C7—H7B	108.2
O1—C4—C5	116.34 (13)	N2—C8—C7	178.0 (2)
N1—C5—C4	113.30 (13)	C6—N1—C5	123.31 (12)
N1—C5—H5A	108.9	C6—N1—H1	118.3
C4—C5—H5A	108.9	C5—N1—H1	118.3
N1—C5—H5B	108.9	C3—O1—C4	106.78 (14)
			
C4—C1—C2—C3	−0.1 (2)	N1—C6—C7—C8	−125.10 (14)
C1—C2—C3—O1	0.5 (2)	O2—C6—N1—C5	−4.5 (2)
C2—C1—C4—O1	−0.39 (19)	C7—C6—N1—C5	175.82 (13)
C2—C1—C4—C5	−179.82 (17)	C4—C5—N1—C6	124.16 (16)
C1—C4—C5—N1	105.9 (2)	C2—C3—O1—C4	−0.7 (2)
O1—C4—C5—N1	−73.48 (17)	C1—C4—O1—C3	0.70 (18)
O2—C6—C7—C8	55.19 (19)	C5—C4—O1—C3	−179.76 (14)

### Hydrogen-bond geometry (Å, º)

**Table:**

*D*—H···*A*	*D*—H	H···*A*	*D*···*A*	*D*—H···*A*
N1—H1···O2^i^	0.86	1.99	2.846 (1)	175
C7—H7*A*···O2^i^	0.97	2.55	3.395 (2)	145

Symmetry code: (i) *x*+1, *y*, *z*.
