# Crystal structure of 2-cyano-*N*-(furan-2-ylmeth­yl)acetamide. Corrigendum

**DOI:** 10.1107/S2056989015013742

**Published:** 2015-07-31

**Authors:** Shivanna Subhadramma, Budanur Papaiah Siddaraju, Chandra Naveen, Janardhanan Saravanan, Dasararaju Gayathri

**Affiliations:** a Department of Physics, Dr M. G. R. Educational and Research Institute University, Maduravoyal, Chennai, India; b Department of Engineering Chemistry, Cauvery Institute of Technology, Sundhahalli, Mandya, India; c Department of Chemistry, Post-Graduate and Research Centre, St. Joseph’s College (Autonomous), Bangalore 560 027, India; d Department of Pharmaceutical Chemistry, PES College of Pharmacy, Hanumanthnagar, Bangalore 560 050, India; e Centre of Advanced Study in Crystallography and Biophysics, University of Madras, Guindy Campus, Chennai 600 025, India

## Abstract

The address of one of the authors in the paper by Subhadramma *et al.* [*Acta Cryst.* (2015), E**71**, o455–o456] is corrected.

## Abstract 2

Corrigendum to *Acta Cryst.* (2015), E**71**, o455–o456.

In the paper by Subhadramma *et al.* (2015) ▸, the address of the first author, Shivanna Subhadramma, was given incorrectly. The correct address should be ‘Department of Physics, Dr M. G. R. Educational and Research Institute University, Maduravoyal, Chennai, India’, and is also given above.
