# Supplementary figures and images for: Crystal structure of 2-cyano-N-(furan-2-ylmeth­yl)acetamide
Source: Acta Crystallogr E Crystallogr Commun. 2015 Jun 10;71(Pt 7):o455–6. doi: 10.1107/S2056989015010488 (PMC4518919; doi:10.1107/S2056989015010488)

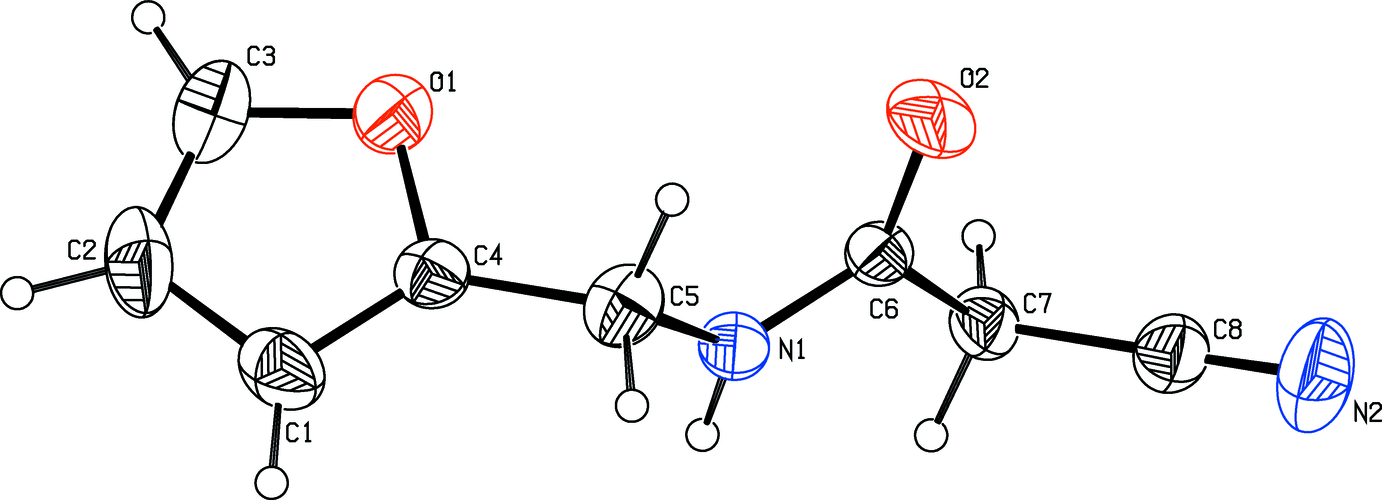

Supplement: Supplementary file 4 [file e-71-0o455-fig1.tif]

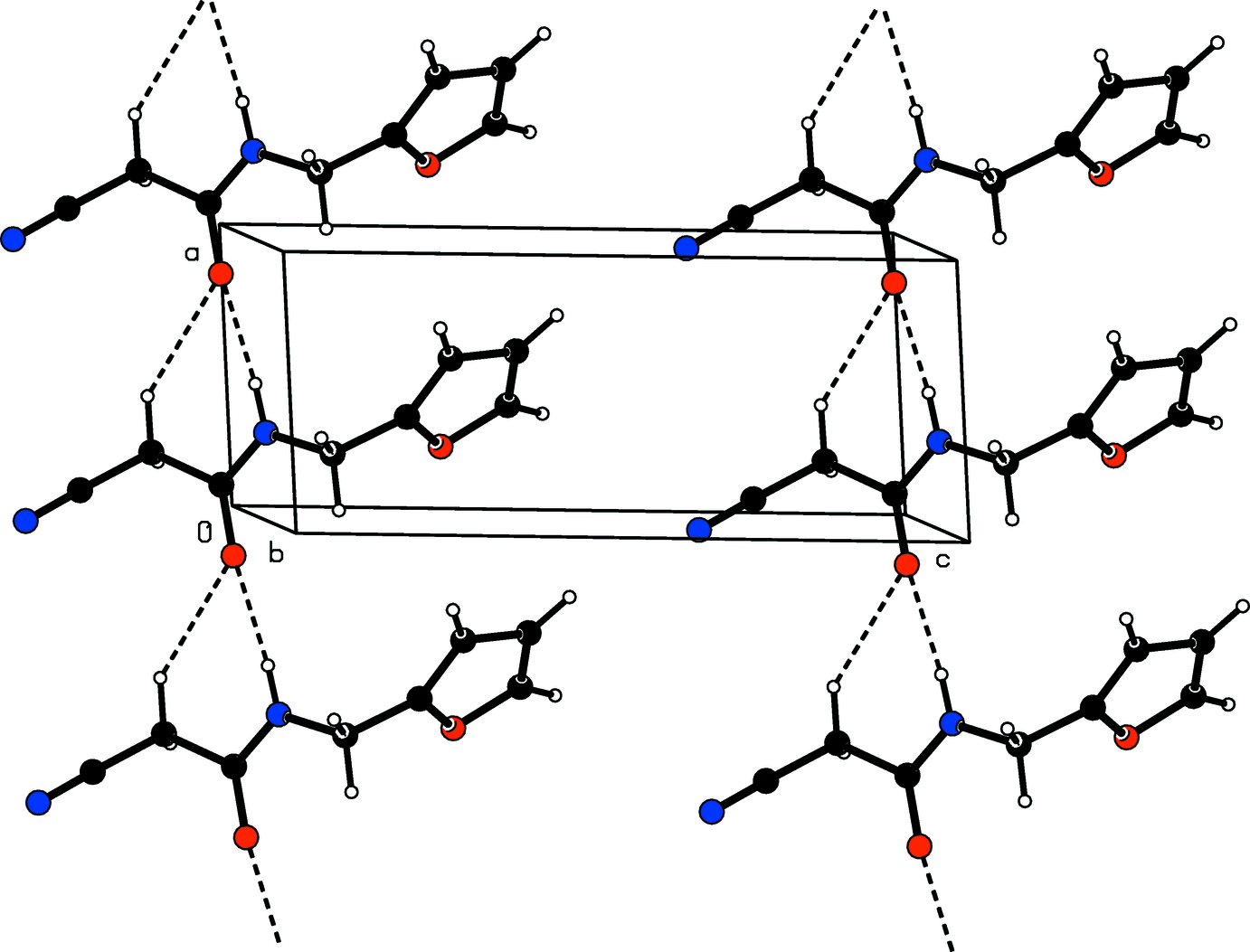

Supplement: Supplementary file 5 [file e-71-0o455-fig2.tif]
